# A Novel Organic–Inorganic Hybrid Admixture for Increasing Flowability and Reducing Viscosity of Ultra-High Performance Paste

**DOI:** 10.3390/ma13153385

**Published:** 2020-07-30

**Authors:** Min Wang, Hao Yao

**Affiliations:** 1 College of Civil Engineering, Key Laboratory for Green & Advanced Civil Engineering Materials and Application Technology of Hunan Province, Key Laboratory of Building Safety and Energy Efficiency of the Ministry of Education, Hunan University, Changsha 410082, China; 2 School of Civil Engineering, Central South University, Changsha 410075, China

**Keywords:** ultra-high performance concrete, polycarboxylate ether, silica fume, admixture, flowability, viscosity

## Abstract

The low flowability and high viscosity of ultra-high performance concrete (UHPC), which is mainly caused by the silica fume (SF) agglomeration and low water–binder ratio, is a severe defect in its engineering applications. Herein, a novel organic–inorganic hybrid (OIH) admixture was synthesized by grafting comb-like polycarboxylate ether (PCE) onto the surface of SF. On the one hand, PCE-grafting could effectively prevent SF agglomeration and improve the dispersion of SF core. The reason being the consumption of polar silicon hydroxyl (Si-OH) groups on the surface of SF and the steric hindrance effect generated from PCE arms. On the other hand, OIH admixture could adsorb onto the surface of cement and SF particles by electrostatic interaction, exhibiting stronger steric hindrance effect than traditional comb-like PCE. As a result, UHPC system with this star-like OIH admixture presented high flowability and low viscosity at low water–binder ratio (0.18).

## 1. Introduction

Ultra-high performance concrete (UHPC) has attracted more and more attention recently because of the high mechanical strength, superior durability, and low permeability. It is understood that these performances of UHPC were caused by the low water–binder ratio (<0.25) and high packing density based on mix designed theoretical principles of concrete [1,2]. In order to conform to designed concrete proportions, large numbers of supplementary cementitious materials such as silica fume (SF), fly ash, blast furnace slag, coal gangue, and gel mud, were employed to occupy the voids between the aggregate particles and cement. It is well-known that the most widely used supplementary cementitious material for UHPC was SF, and its dosage reached up to 15%–30% by weight of cement [3,4,5,6]. As a kind of spherical amorphous particle, SF exhibited micron size, high surface area, and cost-effectiveness. The main composition of SF was SiO_2_ (75–98%), which had high mineral reactivity, and there existed large numbers of polar silicon hydroxyl (Si-OH) groups on the surface of SF [7,8,9]. However, the physicochemical properties of SF and the low water–binder ratio lead to the tempestuous agglomeration of SF and cement particle, resulting in poor workability, i.e., low flowability and high viscosity of UHPC [10,11].

Comb-like polycarboxylate ether superplasticizer (PCE) is the most widely used admixture in cement-based materials, currently, the workability of UHPC is wished to improve by adding comb-like PCE. PCE adsorbed onto mineral phase surface through the synergistic effect of electrostatic repulsion and steric hindrance to disperse cement particles. Unfortunately, in spite of the massive consumption on PCE, the workability of UHPC still was a severe defect in engineering applications [12,13,14,15,16]. The reason was due to the essential difference between UHPC and ordinary concrete, which proposed the different demand for admixture. Specifically, the comb-like PCE enhanced the ordinary concrete workability by effectively improving the dispersion of cement particles, however, the main challenge in UHPC system was how to improve the dispersion of SF [17,18]. Furthermore, the low water–binder ratio of UHPC also brought forward a higher requirement for the dispersibility of PCE. Thus, it is essential to design and synthesize a novel admixture which could be effectively used in UHPC for improving the workability. In principle, one way was to reduce the agglomeration effect of SF, another was to increase the electrostatic repulsion and steric hindrance effect of PCE at low water–binder ratio.

Herein, the organic–inorganic hybrid (OIH) admixture was designed and synthesized by grafting PCE onto the surface of SF. First, the silane coupling agent 3-methacryloxypropyltrimethoxysilane (KH-570) was grafted onto the surface of silica fume (SF) by condensation reaction between SF and KH-570 hydrolysates. Subsequently, the OIH admixture was synthesized by the addition polymerization of KH-570 grafted SF, maleic anhydride (MAH), acrylic acid (AA), and allyl polyoxyethylene glycol ether (APEG). In the structure of OIH admixture, SF particles located at the core of OIH admixture and the traditional comb-like PCE molecules was regarded as the arms. For one thing, the grafting process mitigated the agglomeration of SF which is located at the core of OIH admixture. For another thing, the size effect of star-like OIH admixture exhibited the stronger steric hindrance effect than traditional comb-like PCE which could improve the dispersion of cement and pure-SF particles. As a result, the addition of OIH admixture increased the flowability and reduced the viscosity of UHPC. This work provided not only a new direction for improving the workability of UHPC but also new applications of SF.

## 2. Experimental

### 2.1. Materials

Maleic anhydride (MAH, 99%, Macklin, Shanghai, China), acrylic acid (AA, 99%, Macklin, Shanghai, China), allyl polyoxyethylene glycol ether (APEG, Mn: ~2400, Shandong Bok Chemical Co., Ltd. Zibo, China), ammonium persulfate (APS, 99%, Macklin, Shanghai, China), thioglycolic acid (TGA, 99%, Macklin, Shanghai, China), and 3-methacryloxypropyltrimethoxysilane (KH-570, 98.0%, Macklin, Shanghai, China) were used as received. Comb-like PCE (Mn: ~16782, lab-made) was used as the contrast sample, the solid content was 20%. In this study, PI 42.5 cement and silica fume (SF) were used as cementitious materials. Their chemical compositions are shown in Table 1.

### 2.2. Preparation of the Organic–Inorganic Hybrid (OIH) Admixture

**Figure 1:**
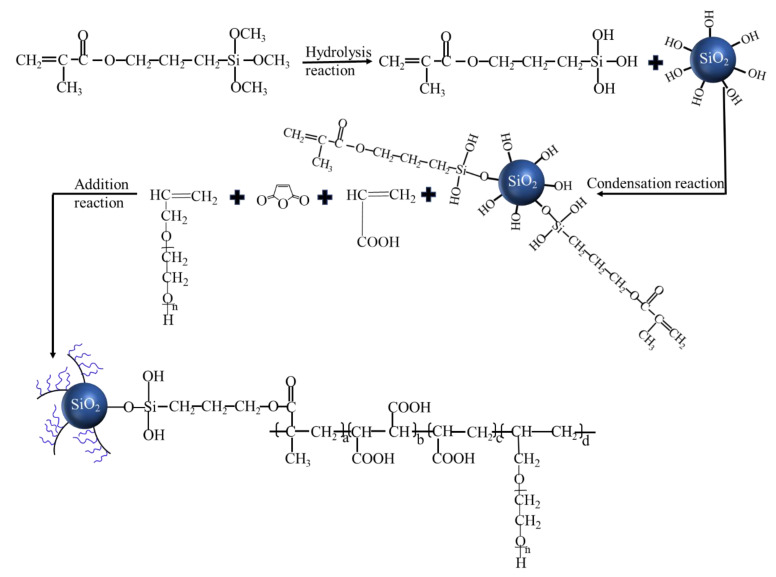
Synthetic route of organic–inorganic hybrid (OIH) admixture.

OIH admixture was synthesized via condensation reaction between KH-570 hydrolysates and SF followed by the addition polymerization of KH-570 grafted SF, MAH, AA, and APEG. The procedure is as follows: first, prescribed amounts of KH-570 and water were charged to the reactor, and then agitation was started for the hydrolysis reaction of Si-O-CH_3_. SF was added to the reaction mixture, and then the hydrolysis product (Si-OH) of KH-570 was reacted with Si-OH from the SF surface. The reaction mixture was stirred at room temperature for 12 h. Second, the aqueous solution of APEG and MAH was added to the reaction mixture at 85 °C. Then, two solutions were prepared whereby one contained TGA and AA, while the second held the APS. These two solutions were fed into the reactor at constant speeds using peristaltic pumps. The solution of AA and TGA was pumped in over 2.5 h, and that the solution of APS was added over 3 h. The mixture was stirred for 2 h at 85 °C after the addition was completed, then cooled to room temperature. After that, OIH admixture was successfully synthesized. Figure 1 exhibited the synthetic route and chemical structure of OIH admixture, in which, SF particles acted as the core and the traditional comb-like PCE molecules acted as the arms. Based on the measurement, the solid content of OIH admixture was 47.5%.

### 2.3. Dispersion Mechanism of OIH Admixture in UHPC System

**Figure 2:**
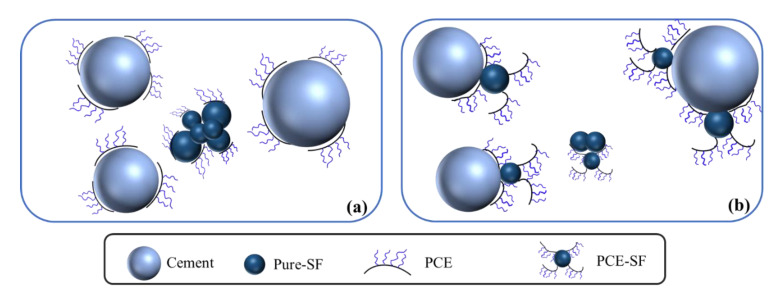
Schematic representation for the dispersion mechanism of (**a**) comb-like polycarboxylate ether (PCE) and (**b**) star-like OIH admixture in ultra-high performance concrete (UHPC) system.

The expected dispersion mechanism of the traditional comb-like PCE and star-like OIH admixture is displayed in Figure 2. As shown in Figure 2a, the traditional comb-like PCE was adsorbed on the surface of cement and SF particles through the electrostatic attraction, while the long chain of polyoxyethylene ether stretched in the pore solution of cement to produce the steric hindrance effect, thus cement particles were dispersed by the synergetic effect of electrostatic repulsion and the steric hindrance. However, the workability of UHPC was still poor, the reason was that the agglomeration of SF particles remained serious and dispersion of cement particles still needed to be further improved at low water–binder ratio. As for OIH admixture in this work, SF particles acted as the core and the comb-like PCE molecules acted as the arms to form a star-like structure, as shown in Figure 2b. First of all, the dispersion of core-located SF improved by the consumption of polar Si-OH groups on the surface of SF and the steric hindrance effect of arm-located PCE. Second, OIH admixture adsorbed onto cement and pure-SF particles by the electrostatic attraction and the size effect of star-like OIH admixture endowed the strong steric hindrance effect in UHPC system, dispersing the particles and preventing them from aggregating. As a result, as an admixture for UHPC, OIH admixture improved the flowability and reduced the viscosity of UHPC.

### 2.4. Mix Design of UHPC Paste Formulation

A simplified model UHPC formulation was used in this research. This recipe included only cement and SF as binder components, while no aggregates or fibers were present. The water–binder ratio was fixed at 0.18, and the binder consisted of 80% cement, 20% SF by weight. It is important to note that SF in this UHPC paste recipe included two parts: One part was core-located SF in OIH admixture; another part was pure-SF without grafting by PCE. The dosage of OIH admixture was from 1 to 5 wt.% (solid content) of binder components.

### 2.5. Characterization of the OIH Admixture

#### 2.5.1. Characterization of the Chemical Structure of OIH Admixture

The obtained OIH admixture was first cleaned through dialysis technique to remove residual unreacted monomers and unsuccessfully grafted PCE. Here, in order to ensure the purity of samples, the conductivity of the dialysate was measured to estimate the content of residue in the dialysate. Dialysis was repeated until the conductivity of the dialysate was almost constant. 

Fourier transforms infrared spectroscopy (FTIR) of pure-SF and OIH admixture samples was recorded by the FTIR spectrometer (NICOLET iS10 IR). To realize the FTIR, after drying, 1 mg purified samples were mixed with 100 mg KBr to make slices.

For X-ray photoelectron spectroscopy (XPS) measurement, 25 mg purified and dried samples were used. XPS analysis was carried out on Kratos Analytical Ltd, (Manchester, UK) and the XPS data were taken on an AXI^S-^Ultra instrument from Kratos Analytical (Manchester, United Kingdom) using monochromatic Al Kα radiation (225 W, 15 mA, 15 kV) and low-energy electron flooding for charge compensation. To compensate for surface charges effects, binding energies were calibrated using C1s peak at 284.80 eV. The data were converted into VAMAS file format and imported into the XPSPEAK software package (XPSPEAK, Hongkong, China) for manipulation and curve-fitting.

#### 2.5.2. Characterization of the Particle Distribution of OIH Admixture

Particle size ranges of pure-SF and OIH admixture were determined using laser particle analyzer (LS 13 320 XR, CA, USA). For particle distribution measurement, SF and OIH admixture were dispersed in water at the same concentration by ultrasonic dispersion.

#### 2.5.3. Micro-Morphologies of OIH Admixture

Micro-morphologies of SF and OIH admixture were observed by Phenom Pro X (Shanghai, China). Pure-SF and OIH admixture were dispersed in water by ultrasonic dispersion at the same concentration (same SF dosage), then dropped onto the silicon slice. The dried samples were coated with gold by sputtering process for excellent electrical conductivity.

### 2.6. Characterization of the Dispersion Mechanism

#### 2.6.1. Adsorption Behavior of OIH Admixture onto Cement and SF Surface

Adsorption behavior of OIH admixture onto cement and SF surface was investigated by X-ray photoelectron spectroscopy (XPS) using an Ultra Axis DLD, XPS (Kratos Analytical Ltd., Manchester, UK) with a Ka radiation source of aluminum and photoelectron energy of 1489.0 eV. Before the XPS measurement, the OIH admixture aqueous dispersion with a concentration of saturated adsorption amount was prepared. After that, cement or SF was mixed with OIH admixture aqueous dispersion in beaker flask and stirred for 2 h, so that OIH admixture was fully adsorbed on the cement and SF particles. The upper suspension was taken out to remove the dissociative OIH admixture, then deionized water was slowly added into the beaker flask; this washing process was repeated five times. The particles were separated by suction filtration, and the obtained lower filter cake dried in a vacuum was used for XPS measurement.

#### 2.6.2. Heat Flow Calorimetry of UHPC Paste

The heat release of reaction for binders was conducted at 20 ± 0.02 °C using TAM Air Calorimeter. Five grams of UHPC paste was added to required water containing specific content of OIH admixture. After mixing for over 2 min at 2500 rpm, the sample was placed in the measuring cell and the heat flow was recorded for 96 h.

### 2.7. Dispersibility of OIH Admixture

#### 2.7.1. Flowability Test

**Figure 3:**
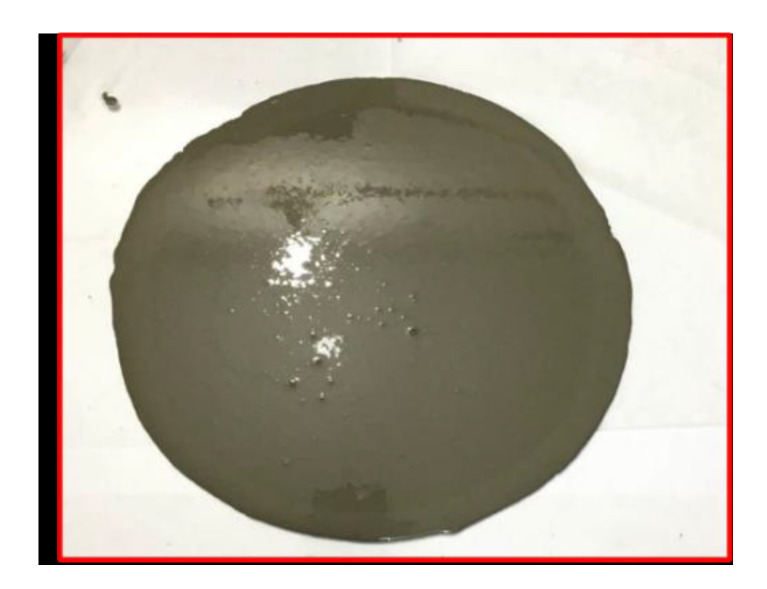
Flowability test result of UHPC paste.

The flowability of UHPC paste was measured by employing a modified mini-slump test according to the Chinese National Standard GB/T 8077–2000. First, cement and SF were preblended in dry form for 200 s. Second, the dry powder material was poured into the water containing the pre-dissolved OIH admixture or comb-like PCE, while the blender was rotating at low speed for 2 min. After resting for 15 s, the paste was mixed for another 2 min at high speed. Then the mixture was poured into a cone mold (base diameter of 60 mm, top diameter of 36 mm, and height of 60 mm) in a clean and moist glass plate. Lifting the mold at about 15 cm above the glass plate would cause the fresh UHPC paste to collapse and spread, as shown in Figure 3. The parallel diameter of the spread was d_1_, and the vertical diameter was d_2_. The value of the (d_1_ + d_2_)/2 is the flowability of UHPC paste. For time-dependent flowability testing, the UHPC paste was put back into the mold and covered with a wet towel after each measurement. Prior to each test, the UHPC paste was again vigorously stirred for 2 min at 285 ± 10 r/min in a planetary mixer. Measurements were performed every 30 min over a period of 120 min, the initial flowability was 5 min after adding the water to the binder mix.

#### 2.7.2. Rheological Behavior Measurement

**Figure 4:**
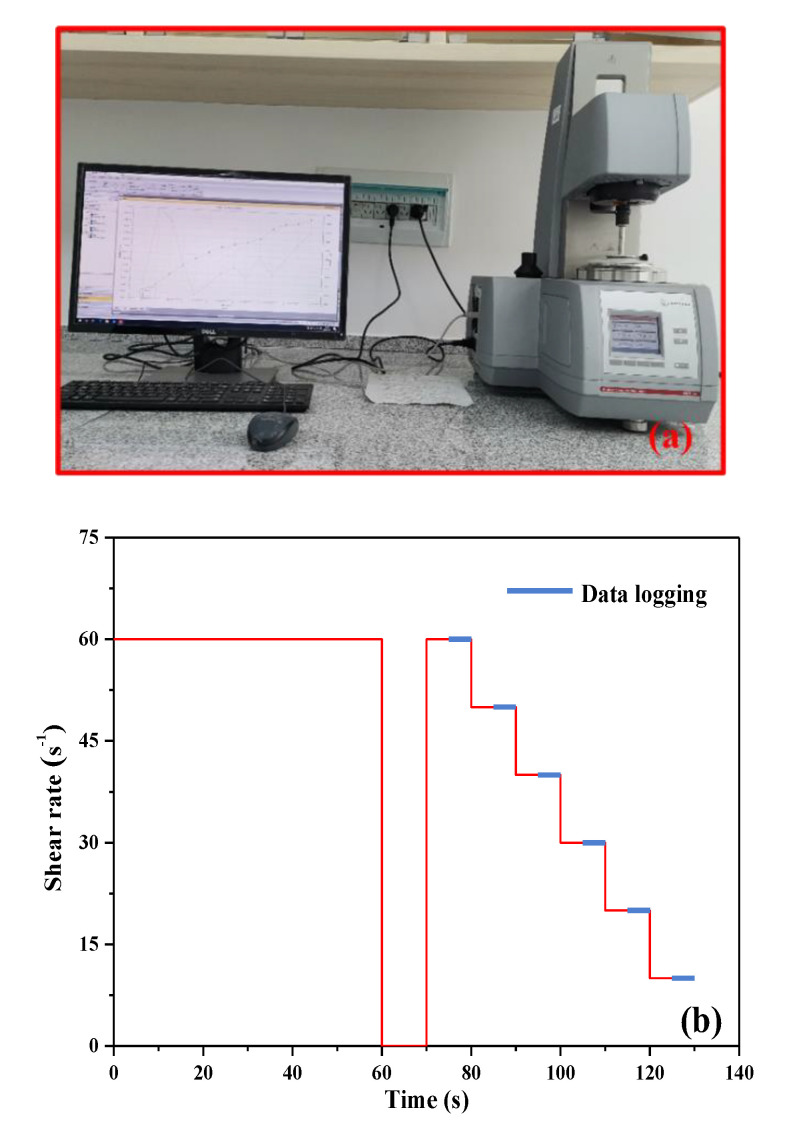
(**a**) Apparatus for rheological test for UHPC paste. (**b**) The testing procedure for measuring rheological parameters of UHPC paste.

Rheological behavior of UHPC paste was measured using a coaxial cylinder rotational rheometer. In order to reduce the slip, the sample cylinder and rotor surface in contact with the UHPC paste were sandblasted. The apparatus and procedure of rheological experiment are shown in Figure 4. After the UHPC paste was prepared, it was poured into the container for measuring. In order to eliminate the effect of initial stress, the paste was sheared at 60 s^−1^ for 60 s, then the sample was left to rest for 10 s. After that, the shear rate was reduced from 60 s^−1^ to 10 s^−1^ through six test sections. Each rotation speed was maintained at 10 s to ensure the paste reached a stable state. For time-dependent rheological test, the UHPC paste was put back into the mixed container and covered with a wet towel after each measurement. Prior to each test, the UHPC paste was again vigorously stirred for 2 min at 285 ± 10 r/min in a planetary mixer. Measurements were performed every 30 min over a period of 120 min.

Herschel–Bulkley equation (Equation (1)) was used to fit and calculate the rheological parameters. Besides, as expressed by Equation (2), using the least square method enables us to calculate the equivalent plastic viscosity (*μ*) in a specific gradient range [19,20,21].

(1) $$T=\tau_{0}+K{\dot{\gamma }}^{n}$$

(2) $$\mu =\frac{3K}{n+2}{\dot{\gamma }}_{max}^{n-1}$$

where τ  is shear stress (Pa), the *τ*_0_ is yield stress (Pa), *K* is consistency coefficient (Pa·s^n^), *n* is dimensionless fluidity index, *μ* is plastic viscosity (Pa·s), and ${\dot{\gamma }}_{max}$ is 60 s^−1^ in this procedure.

#### 2.7.3. Mechanical Properties Measurement

After mixing, samples for flexural and compressive strength tests were prepared by the size of 40 mm × 40 mm × 160 mm for mechanical properties test. The samples were allowed to cure in the mold for 24 h and then curing at room temperature about 20 ℃/RH > 95% for 3 d, 7 d, and 28 d. The flexural and compressive strength were determined using a TYA-300B concrete strength tester.

## 3. Results and Discussion

### 3.1. Characterization of OIH Admixture

**Figure 5:**
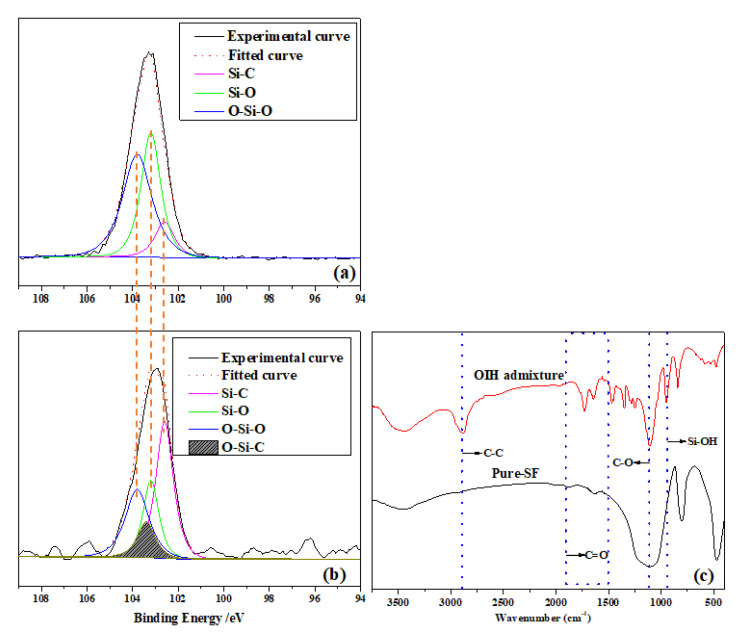
XPS spectra fitted results of Si 2p from (**a**) pure-SF and (**b**) purified OIH admixture. (**c**) FTIR spectra of pure-SF and purified OIH admixture.

XPS is a powerful spectroscopic technique for the characterization of surfaces with chemical bonding. To confirm the successful reaction of the various monomers in the synthesis of OIH admixture as described in Section 2.2, XPS was conducted to investigate the Si 2p (electron sublayer) element composition of the pure-SF and purified OIH admixture. As shown in Figure 5a, Si 2p of pure-SF was fitted three peaks at 102.6, 103.2, and 103.8 eV, which contained the band of Si-C, Si-O, and O-Si-O bond, respectively. However, from Figure 5b, Si 2p of OIH admixture could be separated into four parts, which were assigned to Si-C, Si-O, O-Si-C, and O-Si-O [22,23]. Obviously, KH-570 and SF successfully reacted to generate O-Si-C in the OIH admixture structure, in which the peak was centered at 103.4 eV. Also, the peak area ratio of the Si-C and Si 2p (A_Si-C_/A_Si_) could be used to estimate the amount of Si-C in the whole system. The A_Si-C_/A_Si_ of pure-SF was estimated to be 9.70%, but the same value of OIH admixture was estimated to be 41.60%. This means that the grafting process of PCE onto the SF surface increased the Si-C content in the whole system, resulting from the Si-C in KH-570.

The FTIR spectra of pure-SF and purified OIH admixture are shown in Figure 5c. It can be observed that the FTIR spectrum of purified OIH admixture existed in the representative absorption peak at ~2850 cm^−1^ which belonged to the C–C bond stretching, the peak of C=O bond stretching appeared at 1700~1850 cm^−1^, the peak of C–O accounted for the band at ~1100 cm^−1^. However, these absorption peaks were not detected from the FTIR spectrum of pure-SF, which suggested that PCE was successfully grafted onto the SF surface. Furthermore, the band at ~950 cm^−1^ resulted from the Si-OH bond stretching; it is clearly noticed this band became sharper after the grafting process [24,25,26]. The result indicated the grafting process consumed a large content of Si-OH on the SF surface. Overall, these results proved that the star-like OIH admixture with SF as core and PCE molecules as arm was successfully synthesized.

**Figure 6:**
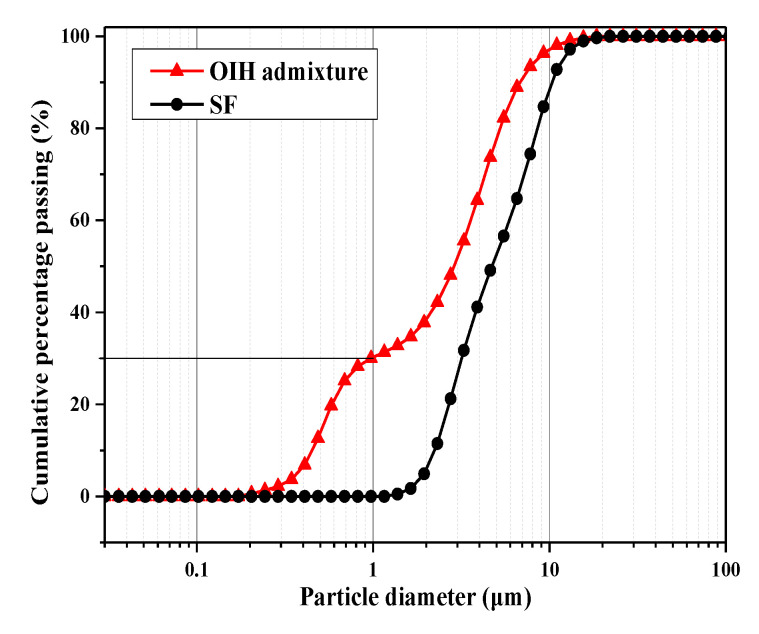
Particle size distributions of pure-SF and OIH admixture.

The particle size distributions of pure-SF and OIH admixture were determined using a laser particle analyzer, the results shown in Figure 6, and reflected the dispersion of SF before and after grafting. Figure 6 represented that the particle size distribution of OIH admixture was smaller than that of pure-SF. The cumulative passing of OIH admixture at 1 μm was 30%, while the corresponding cumulative passing of SF was 0%. The result indicated that the agglomeration of SF reduced after grafting, namely, the grafting of PCE onto SF surface was an effective way to improve the dispersion of SF.

**Figure 7:**
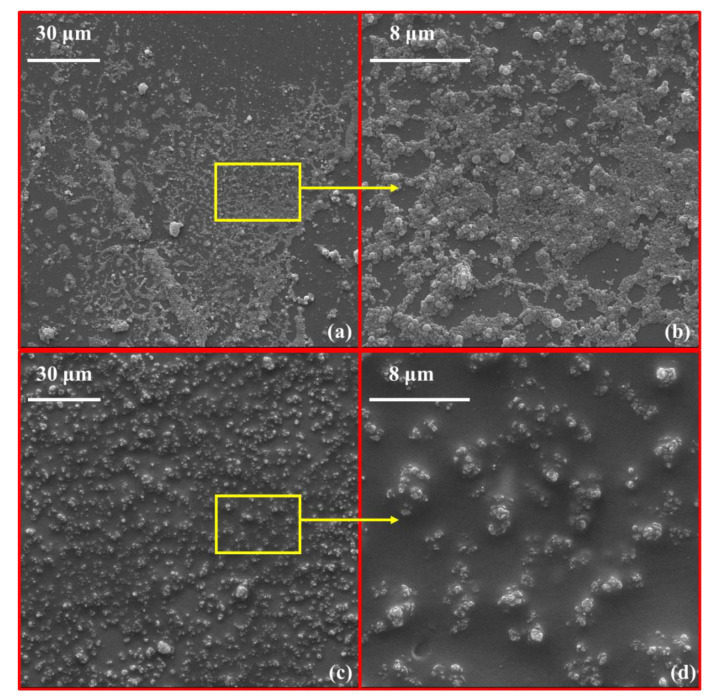
Micro-morphologies of (**a,b**) pure-SF and (**c,d**) OIH admixture by SEM.

Micro-morphologies of pure-SF and OIH admixture were observed by SEM. Figure 7a,b exhibited the micro-morphology of pure-SF; Figure 7c and d represented the micro-morphology of OIH admixture. It can be observed that, compared to the pure-SF, the agglomeration of OIH admixture reduced significantly. The results were in good accordance with the particle size distributions results, which collectively indicated that grafting PCE onto SF effectively improved the dispersion of SF. Moreover, from Figure 7c,d, a lot of organics that appeared around core-located SF can be observed, which played an effective role in the dispersion of SF. As mentioned before, these organics were the traditional comb-like PCE molecules that acted as the arms of OIH admixture. It can be inferred that the arm-located PCE in OIH admixture provided steric hindrance to disperse core-located SF. Also, as discussed above in FTIR spectra of pure-SF and purified OIH admixture, the polar Si-OH groups on SF surface had been consumed after grafting PCE, thereby preventing the coalescence resulting from the condensation reaction of Si-OH between different SF particles [7].

### 3.2. The Adsorption Behavior of OIH Admixture onto Cement and Pure-SF Particles

**Figure 8:**
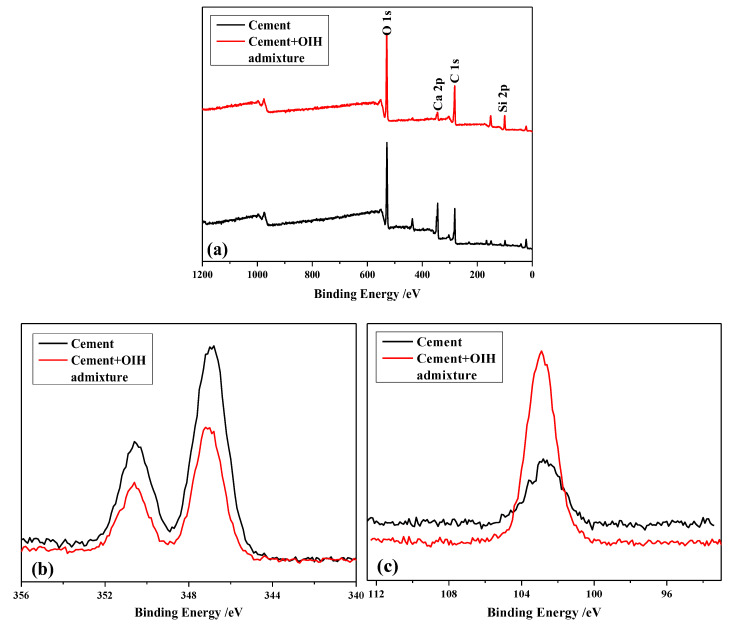
The XPS survey spectra (**a**) wide scan, (**b**) Ca 2p (narrow scan), and (**c**) Si 2p (narrow scan) of cement particles before and after the adsorption of OIH admixture.

As we know, the adsorption of OIH admixture onto the surface of particles is the precondition for dispersibility of OIH admixture. As noted in the wide scan spectra of cement particles before and after the adsorption of OIH admixture (Figure 8a), there were O, Ca, C, and Si elements in their XPS survey spectra. The photoelectron intensity of Ca 2p became weaker after the adsorption of OIH admixture, but the photoelectron intensity of Si 2p became stronger after the adsorption of OIH admixture. The XPS narrow scan spectra of Ca 2p and Si 2p regions are illustrated in Figure 8b and c, respectively. The results show that the photoelectron intensity of Ca 2p decreased after the adsorption of OIH admixture (Figure 8b). It is because that Ca element existed in cement, but not in OIH admixture. OIH admixture covered on cement particles surface after adsorption cause the decrease of Ca 2p photoelectron intensity. However, from Figure 8c, the photoelectron intensity of Si 2p increased after the adsorption of OIH admixture, since OIH admixture contained SF, which introduced large amounts of Si element after adsorption. To conclude, the results from XPS analyses confirmed that OIH admixture presented effective adsorption onto the cement surface in the hydration process.

**Figure 9:**
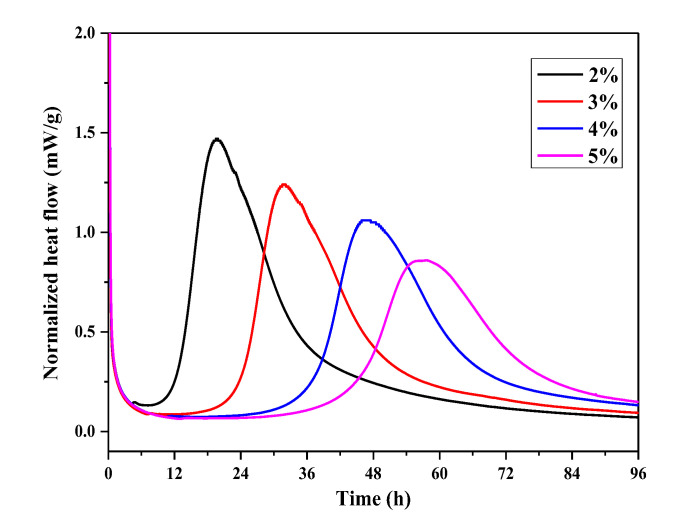
Time-dependent heat flow curves of UHPC paste at different OIH admixture dosage.

In order to further study the adsorption behavior of OIH admixture onto cement, the influence of different dosage OIH admixture on the hydration of the UHPC paste was monitored via isothermal heat flow calorimetry. It is illustrated in Figure 9 that the OIH admixture extended the induction period and depressed hydration peak. The result indicated that OIH admixture retarded the cement hydration. This effect was attributed to OIH admixture adsorption onto cement particles or their hydration products, segregating the cement particles or their hydration products with water. This phenomenon was in good accordance with previous results for the mechanism of PCE on cement hydration [27,28,29]. Moreover, the sample with higher OIH admixture dosage displayed stronger retarded action in all cases. It presented that the dispersion effect strengthened with the dosage of OIH admixture, which resulted from the adsorption behavior of OIH admixture onto the cement particles intensified with OIH admixture dosage.

**Figure 10:**
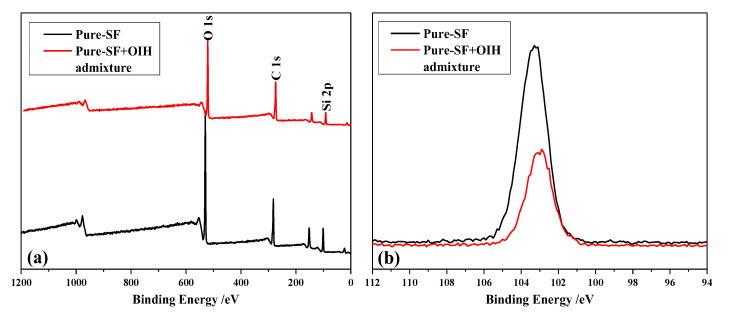
The XPS survey spectra (**a**) wide scan and (**b**) Si 2p (narrow scan) of pure-silica fume (SF) particles before and after the adsorption of OIH admixture.

For the adsorption of OIH admixture on the surface of pure-SF particles, Figure 10 represented the XPS survey curves of the pure-SF particles before and after the adsorption of OIH admixture. According to Figure 10a, there were no new elements introduced into pure-SF after the adsorption of OIH admixture. Apparently, in the case of after adsorption (pure-SF + OIH admixture), the two sources of the Si element were pure-SF and OIH admixture. It should be note that the SF content of two samples of before and after the adsorption of OIH admixture was fixed at preparation. Figure 10b indicated the photoelectron intensity of Si 2p decreased after the adsorption of OIH admixture, which is due to OIH admixture covered on the pure-SF surface after the adsorption.

From the results above, it could be evidenced that OIH admixture effectively adsorbed not only onto the cement particles surface, but also onto the pure-SF particles surface in UHPC system.

### 3.3. Flowability of UHPC Paste

**Figure 11:**
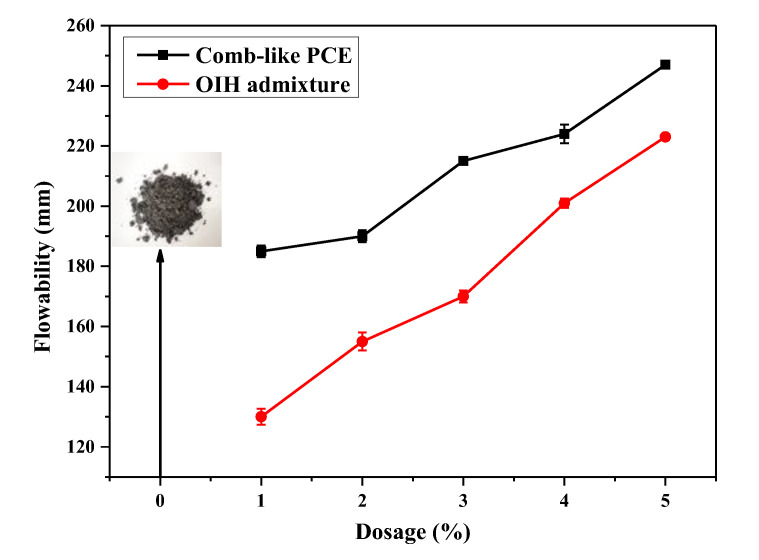
Flowability of UHPC paste at different OIH admixture and comb-like PCE dosage.

The flowability of UHPC paste at different OIH admixture and comb-like PCE dosage was evaluated by a “mini-slump” test. The relationship between flowability of UHPC paste and OIH admixture and comb-like PCE dosage is presented in Figure 11. Also, the initial status of UHPC paste at the water–binder ratio 0.18 is presented in the insert of Figure 11, from which it can be clearly observed that the UHPC paste without OIH admixture and comb-like PCE was of stiff consistency, leading to the severe limitation of UHPC construction. As displayed in Figure 11, the flowability of UHPC paste with comb-like PCE was lower than that of OIH admixture. In the case the dosage was 3 wt.%, the flowability of UHPC paste increased to 215 mm; however, the same value of comb-like PCE was only 170 mm. In other words, as an admixture for UHPC paste, OIH admixture displayed highly effective dispersibility in UHPC paste than comb-like PCE, and the dispersibility increased with the OIH admixture dosage. As mentioned before, the effectively adsorption onto the surface of cement and pure-SF particles was the precondition of the OIH admixture sufficient dispersibility in UHPC system, and the unusual star-like structure was the essential reason for the sufficient dispersibility of OIH admixture in UHPC system.

### 3.4. Flowability Retention Behavior of UHPC Paste

**Figure 12:**
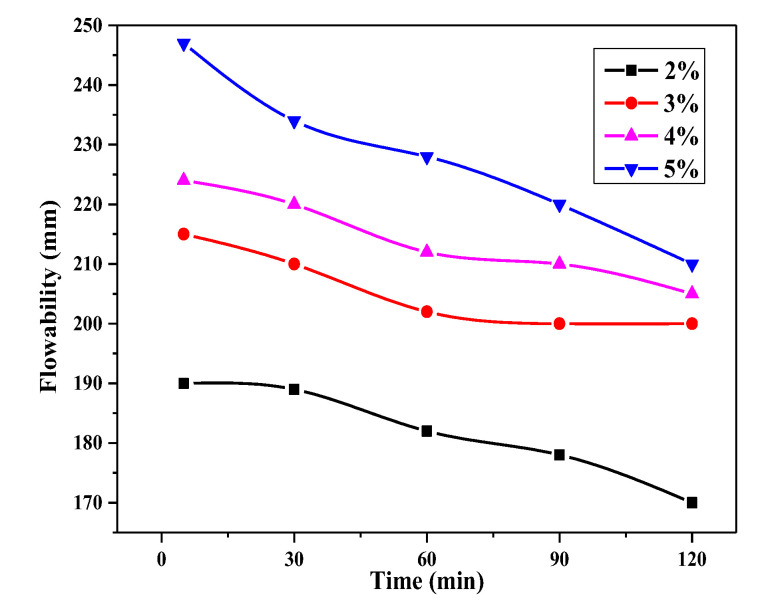
Flowability of UHPC paste with time at different OIH admixture dosage.

Flowability retention of OIH admixture was measured through the flowability loss test of the UHPC paste with time at different dosage. The results are presented in Figure 12. It can be seen that the flowability of UHPC paste decreased with time in all of the dosage. When OIH admixture dosage was higher than 3 wt.%, even loss with time, the UHPC paste also had good flowability, which could assure the construction of UHPC. After 120 min, in the case of OIH admixture dosage was 3 wt.%, the flowability was 200 mm. This desirable flowability retention behavior indicated that the OIH admixture does not quantitatively adsorb onto the cementitious materials surface after it had been added to the UHPC system. In other words, certain portion of OIH admixture still existed in the pore solution and could later adsorb onto the newly formed hydration products, bringing sustainable dispersibility over 2 h.

Moreover, the slope of the flowability retention curve was the flowability loss ratio with time at different dosage. From Figure 12, in the cases of 3 and 4 wt.% OIH admixture dosage, the flowability retention behavior was better than 2 or 5 wt.%. It confirmed that there existed an optimum OIH admixture dosage in UHPC paste for flowability retention behavior. These results can be explained by the fact that OIH admixture in the pore solution was too little at low OIH admixture dosage (2 wt.%) to assure flowability for a long time. On the contrary, at high dosage (5 wt.%), large amounts of OIH admixture in the pore solution could lead to agglomeration of mineral particles, deteriorating the flowability retention behavior.

### 3.5. Rheological Properties of UHPC Paste

**Figure 13:**
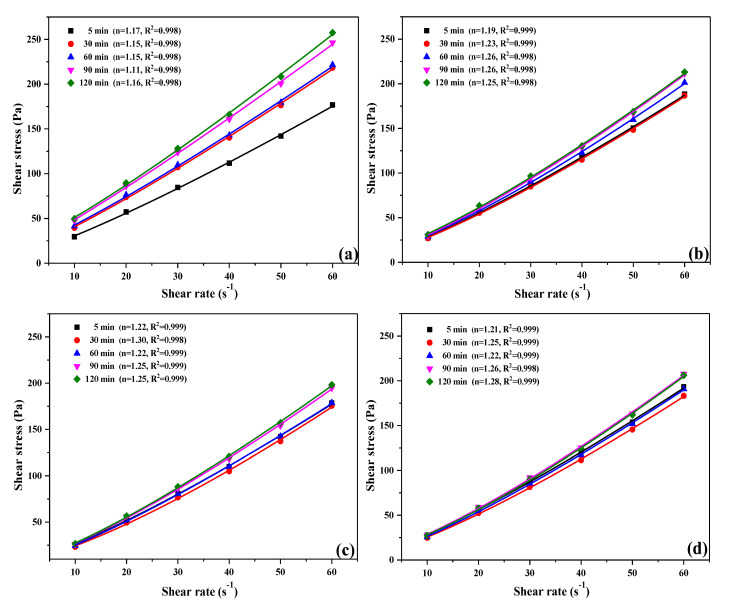
The rheological curves of UHPC paste with time at different OIH admixture dosage (**a**) 2 wt.%, (**b**) 3 wt.%, (**c**) 4 wt.%, (**d**) 5 wt.%.

The rheological curves of UHPC paste with time at different OIH admixture dosage is represented in Figure 13. It is obvious that the fitting coefficients (R^2^) of all samples were above 0.998, it means that the rheological behavior of UHPC paste with OIH admixture was perfectly suitable for the Herschel-Bulkley model. As shown in Figure 13, for all the fitting results with the Herschel–Bulkley model, the fluidity index exceeded 1. It represented that the UHPC paste displayed a slight shear-thickening response at all OIH admixture dosage. The shear-thickening behavior almost heightened with the increase of OIH admixture dosage. It can be explained by the fact that the rise of shear rate could lead to separation of cementitious particles with different specific surface and size, aggravating the cohesion of SF particles with large specific surface and small size, which resulted in the higher viscosity of the UHPC system. Moreover, the increase of shear rate exacerbated the coalescence of OIH admixture in the pore solution by desorbing, which might lead to an increase in the viscosity of the UHPC paste with the shear rate [30].

**Figure 14:**
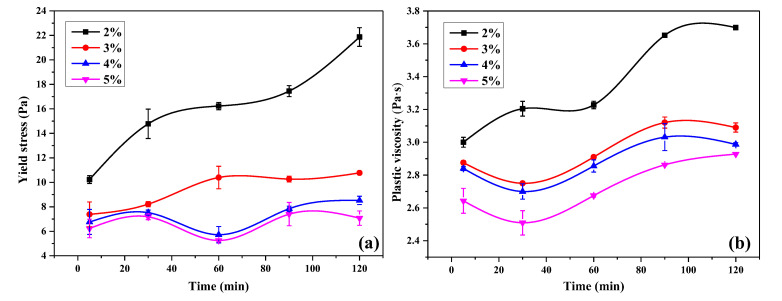
(**a**) Yield stress and (**b**) plastic viscosity of UHPC paste with OIH admixture dosage at different time.

The relationship between the yield stress of UHPC paste which was calculated by the Herschel-Bulkley model is plotted in Figure 14a. In the case of OIH admixture dosage was 3 wt.%, the yield stress of UHPC paste was lower than that of the case of 2 wt.% at all time. The reduction of yield stress was caused by the segregation of the paste with OIH admixture. After grafting PCE onto the surface of SF, the particle coalescence of core-located SF was reduced, and the adsorption of OIH admixture onto the cement and pure-SF particles resulted in the drop of flocculation. The yield stress of UHPC paste almost remained unchanged after the dosage of OIH admixture larger than 3 wt.% (4 and 5 wt.%), it represented that the dispersion function of OIH admixture for UHPC paste reached saturation.

Figure 14b set out the plastic viscosity of UHPC paste as a function of time. It can be seen from Figure 14b that the plastic viscosity of UHPC paste almost reduced with the improvement of OIH admixture dosage at the same time. The result indicated that the OIH admixture had effective dispersibility which could be utilized to reduce the viscosity of UHPC paste. Also, when the dosage was higher than 3 wt.%, the plastic viscosity of UHPC paste barely aggravated with time. That is, OIH admixture had good time stability effect on the UHPC paste viscosity.

### 3.6. Mechanical Properties of UHPC

**Figure 15:**
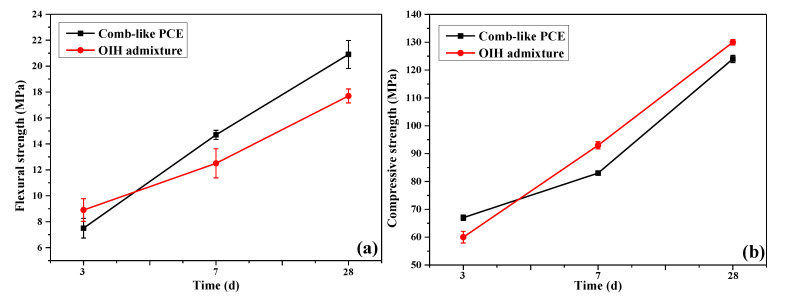
(**a**) Flexural strength and (**b**) compressive strength of UHPC paste with OIH admixture and comb-like PCE at different curing age.

In order to reach the same flowability for UHPC paste, the dosage required for comb-like PCE and OIH admixture was 3.8% and 2.0%, respectively. At the same flowability, the flexural and compressive strength test results of UHPC with comb-like PCE and OIH admixture are compared in Figure 15a,b. At 3 d, the flexural and compressive strength of UHPC with OIH admixture were lower than that of comb-like PCE, which was due to the strong retardation effect of OIH admixture on UHPC hydration. However, at 7 d and 28 d, it is clear that UHPC with OIH admixture had higher flexural and compressive strength compared to UHPC with comb-like PCE. OIH admixture improved the dispersion and prevented the agglomeration of SF; the satisfactory dispersion of SF strengthened the filling effect on UHPC system, then improving the flexural and compressive strength of UHPC. 

## 4. Conclusions

In the present work, the organic–inorganic hybrid (OIH) admixture was successfully synthesized through the condensation reaction between SF and KH-570 hydrolyzates, followed by the addition polymerization of KH-570 grafted SF, MAH, AA, and APEG. The synthesized OIH admixture possessed a unique star-like structure, in which, the comb-like PCE molecules acted as the arms and the SF particles served as the core. After PCE grafting, the consumption of surface polar Si-OH groups and the steric hindrance effect generated from arm-located PCE jointly improved the dispersion of core-located SF. Moreover, star-like OIH admixture adsorbed onto the cement and SF particles by electrostatic repulsion which exhibited the strong steric hindrance effect and hence prevented the cement and SF particles from agglomerating. As a result, OIH admixture presented sufficient dispersibility as an admixture for UHPC. The flowability of UHPC paste increased with OIH admixture dosage when the water–binder ratio was 0.18, and the optimum OIH admixture dosage was 2 or 3 wt.% for flowability retention behavior. The yield stress and plastic viscosity of UHPC paste reduced after the addition of OIH admixture, it also showed the good time stability effect on the UHPC paste viscosity. The star-like OIH admixture had significant improvement on the workability of UHPC. The flowability, yield stress, plastic viscosity, setting time, and mechanical properties of UHPC can also be adjusted by controlling the category and density of branched-chains on star-like structure. Also, the telechelic and hyperbranched structure could be used in designing and synthesis of chemical admixture, which will be studied in further work.

## Figures and Tables

**Table 1:** Chemical compositions of the cement and silica fume.

Material	Mass Fraction/wt.%								
	SiO_2_	Al_2_O_3_	Fe_2_O_3_	CaO	MgO	SO_3_	Na_2_O	K_2_O	Loss
**Cement**	22.87	4.47	3.48	64.05	2.46	2.44	0.52	0.9	1.21
**Silica fume**	95.38	-	0.61	1.84	0.26	-	0.16	0.85	2.48
